# An Aurora Kinase B–Based Mouse System to Efficiently Identify and Analyze Proliferating Cardiomyocytes

**DOI:** 10.3389/fcell.2020.570252

**Published:** 2020-10-07

**Authors:** Wenbin Fu, Qiao Liao, Liangpeng Li, Yu Shi, Andi Zeng, Chunyu Zeng, Wei Eric Wang

**Affiliations:** ^1^Department of Cardiology, Daping Hospital, The Third Military Medical University, Chongqing, China; ^2^Department of Cardiology, Daping Hospital, Army Medical University, Chongqing, China; ^3^Chongqing Institute of Cardiology, Chongqing, China; ^4^Key Laboratory of Myocardial Ischemia, Ministry of Education, Harbin Medical University, Harbin, China; ^5^Cardiovascular Research Center, Chongqing College, University of Chinese Academy of Sciences, Chongqing, China

**Keywords:** Aurora kinase B, mice, myocardium, development, regeneration

## Abstract

To identify and analyze the live proliferating cardiomyocytes is crucial for deciphering the mechanisms controlling endogenous cardiac regeneration. Traditional methods confuse cell division with multinucleation in postnatal cardiomyocytes. Recent efforts have achieved significant progress on discerning cytokinesis from only nuclear division. However, those methods were either designed to label post-cytokinesis progeny or challenging to sort the live proliferating cardiomyocytes. In this study, we highlighted an Aurora kinase B reporter–based mouse system with a tdTomato fluorescence labeling. It could efficiently identify proliferating cardiomyocytes in neonates. The analysis of sorting tdTomato^+^ cardiomyocytes with different ploidy indicated that mononucleated cardiomyocytes might not possess significantly higher proliferating potential than other cardiomyocytes when most cardiomyocytes have become post-mitotic. Moreover, tdTomato^+^ cardiomyocytes were significantly increased and enriched at injury border zone after apex resection in neonates, while there were no increased tdTomato^+^ cardiomyocytes after myocardial infarction in adults.

## Introduction

Myocardial infarction (MI) is among the leading causes of morbidity and mortality worldwide, severely affecting human health (Waks and Buxton, 2018). MI leads to a rapid loss of a large number of cardiomyocytes, resulting in irreversible heart failure. Current treatments for MI, such as thrombolysis, percutaneous coronary intervention, or coronary artery bypass grafting, can recanalize blocked coronary arteries. Drug therapy can alleviate cardiac remodeling, but cannot fundamentally repair dead cardiac tissue (Cahill et al., 2017). Thus, new therapeutic approaches for regenerating lost myocardium to reverse heart failure are strongly needed.

In recent years, the fate-mapping technique has proved that the primary source of endogenous cardiac regeneration is the proliferation of pre-existing cardiomyocytes (Senyo et al., 2013; He et al., 2017; Nakada et al., 2017). It is known that adult mammalian cardiomyocytes can proliferate but at a meager rate (Bergmann et al., 2015). Under the stimulation of MI injury and other factors, adult cardiomyocytes can reenter the cell cycle but cannot accomplish cytokinesis (Mohamed et al., 2018). To identify and analyze the live proliferating cardiomyocytes is crucial for deciphering the mechanisms controlling endogenous cardiac regeneration. One of the significant limitations of identifying proliferating cardiomyocytes is the evaluated method of cardiomyocyte proliferation. The complete process of cardiomyocyte proliferation includes DNA replication, mitosis, and cytokinesis (Broughton and Sussman, 2019). The incomplete cell division leads to the formation of binuclear or multinuclear cardiomyocytes, which does not increase the cell numbers (Alkass et al., 2015). The traditional methods mainly focused on the markers of DNA replication and nuclear division, such as Ki67, BrdU/EdU, and PHH3, which poorly differentiates authentic cell division from endoreduplication, cytokinetic mitosis, or DNA repair (Hesse et al., 2012; Cai et al., 2018; Wodsedalek et al., 2019).

Aurora kinase B (Aurkb) controls the movement and separation of chromosomes by combining specialized microtubules called K-fibers and gathering in the equatorial plate at the end of cell division. It controls microfilaments’ contraction to constrict cleavage furrow, which gradually deepens so that the cell finally splits into two (Murata-Hori et al., 2002; Goldenson and Crispino, 2015). Although Aurkb is an essential indicator of cell division and used in most studies to describe cardiac regeneration, the cytokinesis time of cardiomyocytes is transient. A single Aurkb marker is challenging to detect in such a short time window. Microscopically, the staining of Aurkb presents a point between symmetrical cells that is difficult to distinguish from non-specific staining, which causes functional specificity but low sensitivity of this method.

Therefore, we generated a mouse system using the Aurkb gene as a non-fusion fluorescent protein signal to mark the proliferating cells, which specifically amplified the transcriptional message of Aurkb gene and efficiently labeled and sorted proliferating cells. Besides, we also provided the quantification and distribution of proliferating cardiomyocyte in healthy and diseased hearts, which was valuable to explore new findings of cardiac regeneration.

## Materials and Methods

### Generation of Transgenic Mice

All mouse studies were carried out in strict accordance with the guidelines of the Institutional Animal Care and Use Committee at Third Military Medical University. The *Aurkb-rox-tdTomato* knock-in mouse line was generated by knocking *rox–Stop–rox–kozak–tdTomato–WPRE–polyA–Frt–Neo–Frt* into the site encoding the translational start codon (ATG) in Aurkb gene. Two arms on the 5′ and 3′ sides of the first coding exon of the Aurkb gene were generated in targeting vectors by homologous recombination. The targeting vector was linearized with I-CeuI digestion and then electroporated into C57 ES cells. The targeting vector containing the previously mentioned cassettes was knocked into the Aurkb locus for endogenous expression of tdTomato cDNA. After drug screening by G418 and Ganc, 144 clones were selected for DNA retrieval and a further selection of positive clones. Long polymerase chain reaction (PCR) assays with primer pairs spanning the targeting vector and flanking genomic DNA were performed to identify neomycin-resistant clones. After verification of correct karyotype, eight positive ES clones were microinjected into blastocysts. The resulting chimeric mouse lines were then crossed to C57BL/6J lines for germline transmission. The correct establishment of *Aurkb-rox-tdTomato* knock-in mouse lines was confirmed by PCR analysis. The *CAG–Dre*, and *Tnnt2–Dre* knock-in mouse lines were generated by genome editing using CRISPR–Cas9 technology and gifted by Pro. Bin Zhou at the Shanghai Institute of Biochemistry and Cell Biology (He et al., 2017).

### Genomic PCR

Genomic DNA was extracted from mouse tails. Tissues were incubated overnight at 55°C with lysis buffer containing 100 mM Tris HCl (pH 7.8), 5 mM EDTA, 0.2% SDS, 200 mM NaCl, and 100 μg/mL proteinase. After centrifugation at 12,000*g* for 10 min, supernatants with genomic DNA were collected. DNA was precipitated by isopropanol, washed in 70% ethanol, and dissolved in deionized water. All mice were genotyped using genomic PCR with the primer listed in Supplementary Table 1.

### Apical Resection in Neonatal Mice

Neonatal mice (1 day after birth) were subjected to apical resection, as described previously (Mahmoud et al., 2014). Briefly, all pups were transferred from the nursing mother to a clean cage; the separated time from the mother should be minimized to reduce the risk of maternal cannibalization. One by one, pups were placed on an ice bed for 3 min to achieve hypothermia anesthesia, which was confirmed by observing apnea and akinesia, and then transferred to the surgical area in a supine position. Thoracotomy was performed along the fourth intercostal area, and the heart apex was exteriorized by gentle pressure on the abdomen. Approximately 15% of the left ventricle was resected using iridectomy scissors. We closed the ribs and muscles together using 7-0 sutures and sealed the skin incision using skin glue. The sham manipulation was performed followed the same procedure except for apical resection. After warming for 3 min and adaption in the mother’s bedding for 30 min, pups were returned to the mother’s nest.

### Myocardial Infarction in Adult Mice

Adult mice (8 weeks of age) were subjected to surgical manipulation as described previously (Yang et al., 2017; Yue et al., 2017). Briefly, mice were endotracheally intubated and anesthetized with 2% isoflurane gas connected to the ventilator. After disinfecting chest with iodine, a horizontal 2-cm incision along the underarm plane was made at the chest skin. The muscle and fascia were bluntly dissected to expose intercostal spaces, and then a thoracic protractor was placed between the third and fourth intercostal ribs. After presenting the internal chest, a permanent ligation was performed at the left anterior descending artery (LAD) with a 7-0 suture. Successful ligation was judged by the fast-changing signs of pale and cyanosis. The incision was then closed in layers of ribs, muscle, and skin with 5-0 sutures. After disinfecting surgical incision with iodine, the mice were sequentially supported with mechanical ventilation and pure oxygen until they resumed spontaneous breathing. The sham manipulation was performed followed by the same procedure except for LAD ligation.

### Cardiomyocyte Isolation

Neonatal (7 days after birth) cardiomyocytes were isolated as described previously (Mahmoud et al., 2014). Briefly, mice were intraperitoneally injected with 100 μL heparin (6.25 U/μL) to prevent clotting. Thirty minutes later, the mice were anesthetized with 2% isoflurane, and then dissected hearts were ligated to a Langendorff perfusion system through the aortic cannula. The hearts were firstly perfused with perfusion buffer (140 mM NaCl, 4 mM KCl, 1 mM MgCl_2_, 10 mM HEPES, 10 mM taurine, 10 mM 2,3-butanedione monoxime and 10 mM glucose, pH 7.3) for 5 min to remove residual blood and secondly digested with digestion buffer (perfusion buffer containing 1 mg/mL collagenase II, 0.12 mg/mL trypsin and 0.02 mM CaCl_2_) for 12–15 min until the hearts became softened and collapsed. The digested hearts were transferred to a dish, minced by scissors, and then dispersed to the cell suspension. The stop buffer (perfusion buffer containing 5 mg/mL bovine serum albumin and 0.1 mM CaCl_2_) was added to the suspension to terminate the digestion. The isolated cells were filtered through a 100-μm strainer and centrifuged for 3 min at 50*g* to collect cardiomyocytes. The cardiomyocytes with different ploidy were identified by nuclear staining (NucBlue Live, Invitrogen, R37610) and isolated using pipettes.

### Cell Cycle Analysis

Neonatal (within 24 h after birth) cardiomyocytes were isolated as described previously (Wang et al., 2017). Briefly, the hearts were removed after 2% isoflurane-induced anesthesia and placed in pre-cold 1 × Hanks solution. After washing and removing auricular appendage, the ventricle tissues were cut into pieces and digested by 0.01% trypsin and 0.08% collagenase II. The cell suspension was collected and pre-plated with 89% Dulbecco modified eagle medium, 10% fetal bovine serum, and 1% penicillin–streptomycin for 1 h to remove a large proportion of non-cardiomyocytes. The remaining cell suspensions were subjected to fluorescence-activated cell sorting and cell cycle analysis using a Cell Cycle Assay Kit (Fluorometric-Green, Abcam, ab112116) according to the manufacturer’s instructions. The cardiomyocytes were prepared at a density of 1 × 10^6^ cells/mL and incubated with culture medium containing Nuclear Green CCS1 for 60 min in a 37°C, 5% CO_2_ incubator. After washing three times with culture medium, the cardiomyocytes were centrifuged at 300*g* for 5 min and resuspended in assay buffer. For *Tnnt2-Dre* × *Aurkb-rox-tdTomato* mice, cardiomyocytes were additionally defined by the expression of cTnT (BD Pharmingen, 565744). The fluorescence intensity was monitored by a BD FACSAria^TM^ III (BD Biosciences, San Jose, CA, United States). The percentages of cells in the G1, S, and G2-M phases were determined.

### Live Cell Imaging

To capture the division events of live cardiomyocytes *in vitro*, we carried out long-term time-lapse microscopy using an Olympus IX83 inverted microscope with a humidified cell culture chamber in the presence of 5% CO_2_ at 37°C. Neonatal (within 24 h after birth) cardiomyocytes were isolated from *CAG-Dre* × *Aurkb-rox-tdTomato* or *Tnnt2–Dre* × *Aurkb–rox–tdTomato* mice. At the initial culture stage, 100 fields of 10 × objective lens were randomly selected, and the absolute coordinate was recorded in the microscopy software. The time-lapse images were taken at intervals of 30 min for a total of 5 days. Time-lapse movies were generated and exported using CellSens Dimension software (Olympus version 1.14, Japan).

### Immunostaining

Hearts were rinsed with phosphate-buffered saline (PBS) and then fixed in 4% paraformaldehyde at 4°C overnight. After three washes in PBS, the heart tissues were in turn dehydrated in 15 and 30% sucrose in PBS solution at 4°C overnight and then embedded in optimum cutting tissue (O.C.T., Sakura); 10-μm-thick cryosections were performed and stored at −20°C until use. For immunostaining, tissue sections were immersed in PBS for 30 min to remove O.C.T., permeabilized with 0.1% Triton X-100, and then blocked with 2.5% normal donkey serum in PBS for 30 min at room temperature. Sections were incubated with the primary antibody overnight at 4°C. The next day, these sections were washed with PBS three times and incubated with Alexa-Fluor–conjugated secondary antibodies (Invitrogen) for 1 h at 37°C. DAPI (4′,6-diamidino 2-phenylindole) was used for nuclear counterstaining.

Antibodies to the following proteins were used: tdTomato (Rockland, 600-401-379; 1:200 dilution; 0.005 μg/μL), Tnni3 (Abcam, ab56357; 1:100 dilution; 0.01 μg/μL), cTnT (Invitrogen, MA5-12960; 1:100 dilution; 0.01 μg/μL), PECAM (BD Pharmingen, 553370; 1:100 dilution; 0.01 μg/μL), α-SMA (Abcam, ab7817; 1:100 dilution; 0.01 μg/μL), and Col1 (Invitrogen, MA1-26771; 1:100 dilution; 0.01 μg/μL). Images were captured by using an Olympus confocal laser scanning microscope (FluoView 3000, Japan) and calculated using the Image Pro Plus 6.0 software.

### Wheat Germ Agglutinin Staining

The cellular outline was marked using wheat germ agglutinin (WGA) staining (Invitrogen, W11261, W32466). After immunostaining, heart sections were incubated with Alexa Fluor 488– or 647–conjugated WGA for 10 min at room temperature according to the manufacturer’s instructions.

### EdU Detection

For EdU (5-ethynyl-2′-deoxyuridine, Thermo Fisher Scientific, A10044) pulse-chase experiment *in vivo*, animals were injected intraperitoneally at 100 μg per animal in P3 and P5. The hearts were harvested at P7. EdU staining was performed using a Click-iT Plus EdU Alexa Fluor 488 Imaging Kit (Thermo Fisher Scientific, C10637), according to the manufacturer’s instructions.

### Quantitative Real-Time Polymerase Chain Reaction

Total RNA was isolated from cells using Trizol reagent (Takara, 9109) according to the manufacturer’s protocols. One microgram RNA per sample was reversely transcribed into cDNA using PrimeScript RT Master Mix (Takara, RR036A) with random primers. Three duplicates with cDNA and SYBR Premix EX Taq^TM^ II (Takara, RR820A) were performed in CFX96^TM^ Real-Time PCR Detection System (Bio-Rad, United States). PCR was conducted in a 10 μL reaction system. Reversed transcription was performed at 37°C for 15 min, and cDNA was amplified for 39 cycles: 95°C for 10 s, 58°C for 20 s, and 72°C for 10 s. Values were normalized to GAPDH to calculate the relative RNA expression levels. The primer sequences used to detect mRNA expression are listed in Supplemental Table 1.

### Western Blot Analysis

For Western blot analysis, the total proteins of samples were isolated by RIPA lysis buffer. The protein samples were prepared with a 5 × sample loading buffer and resolved on sodium dodecyl sulfate–polyacrylamide gel electrophoresis gels. Transferred NC membranes were probed with antibodies against Aurkb (Abcam, ab2254, 1:1,000, 0.001 g/μL) and GAPDH (Proteintech, 10494-1-AP, 1:1,000, 0.001 g/μL). Chemiluminescent signals were detected by the LiCor Odyssey Fc instrument.

### Statistical Analysis

Statistical analyses were performed using GraphPad Prism 6.0 for Windows (GraphPad Software, 20 San Diego, CA, United States). All numeric data in this experiment are presented as the mean ± standard error (mean ± SE). For comparison of more than two groups, statistical analyses were performed by one-way analysis of variance followed by Tukey multiple comparisons. For comparison of two groups, statistical analyses were conducted by the Student *t* test. *P* < 0.05 was considered as statistically significant.

## Results

### The Proliferating Cells Were Efficiently Labeled in *CAG-Dre* × *Aurkb-rox-tdTomato* Neonates

**FIGURE 1:**
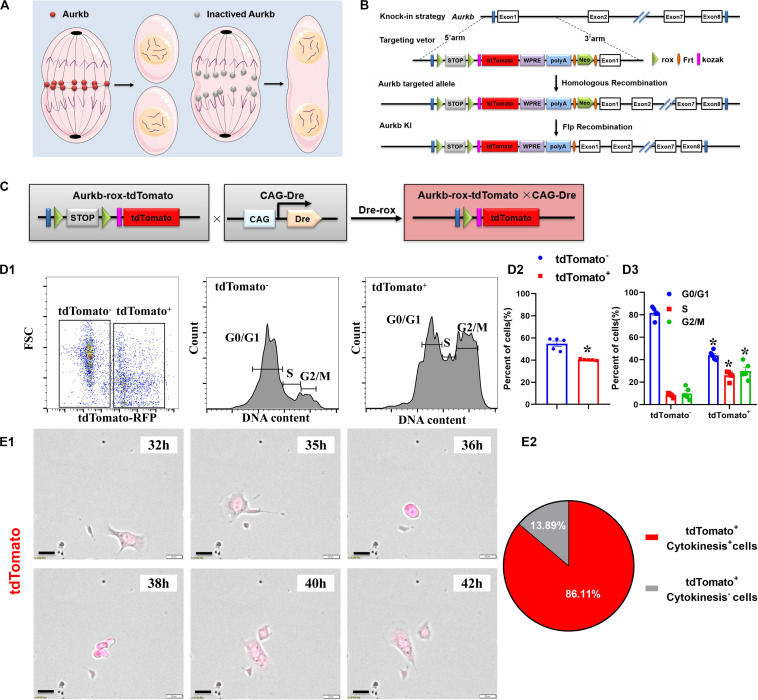
The proliferating cells were efficiently labeled in *CAG-Dre* × *Aurkb-rox-tdTomato* neonates. **(A)** Schematic figure showing Aurkb’s necessity for cell proliferation. **(B)** Schematic figure showing knock-in strategies for *Aurkb-rox-tdTomato* alleles by homologous recombination. **(C)** *CAG-Dre* mice were used to cross with *Aurkb-rox-tdTomato* mice to label all proliferating cells. **(D)** Representative images of flow cytometry analysis **(D1)** and quantification of tdTomato^±^ cells **(D2)** and numbers in G1, S, G2/M phases **(D3)** from ventricles of P1 *CAG-Dre* × *Aurkb-rox-tdTomato* mice. *n* = 5. *p < 0.05 vs. tdTomato^–^. **(E)** Representative images **(E1)** and quantification **(E2)** of time-lapse imaging analysis show cell proliferation *in vitro* of tdTomato^+^ cells from the ventricles of P1 *CAG-Dre* × *Aurkb-rox-tdTomato* mice. Scale bar = 20 μm.

Activated Aurkb is necessary for cell proliferation (Figure 1A). Based on the *Dre-rox* system, we generated an Aurkb transcription–driven un-fusion red fluorescent protein (*Aurkb-rox-tdTomato*) under the control of endogenous Aurkb locus to reflect proliferative potential in mouse heart (Figure 1B). *CAG-Dre* mice were used to cross with *Aurkb-rox-tdTomato* mice to label all proliferating cells (Figure 1C). As expected by the design of this recombination system, immunostaining of sections in P4 neonatal hearts showed that expression of tdTomato in *CAG-Dre* × *Aurkb-rox-tdTomato* mice but not of tdTomato was activated in the *Aurkb-rox-tdTomato* mice (Supplementary Figure 1A). Because the un-fusion of red fluorescent protein was engineered, a whole-cytoplasmic and specific expression of tdTomato could be observed in P56 heart sections when marking cellular outline using WGA staining (Supplementary Figure 1B). Moreover, qPCR results indicated that genetic labeling did not influence the transcriptional level of Aurkb (Supplementary Figure 2A).

To determine the labeling efficiency of tdTomato on cell proliferation, we monitored changes in the cell cycle by flow cytometry distinguished by the positive and negative expression of tdTomato. The protein expression of Aurkb was confirmed to be significantly increased in sorted tdTomato^+^ cells than sorted tdTomato^–^ ones (Supplementary Figure 2B). Moreover, in isolated cells from P1 ventricles of *CAG-Dre* × *Aurkb-rox-tdTomato* mice, the percent of tdTomato^+^ cells was 40.30% ± 0.29%, and tdTomato^+^ cells exerted decreased cell numbers in G1 phase and increased cell numbers in the S and G2/M phases (Figure 1D). In *in vitro* experiments, the complete proliferation process of cells isolated from the ventricles of P1 *CAG-Dre* × *Aurkb-rox-tdTomato* mice was identified with time-lapse imaging observation as a “gold standard” method. It showed that 86.11% of the tdTomato^+^ cells underwent a complete proliferation process (Figure 1E and Supplementary Video 1). These results suggested that *CAG-Dre* × *Aurkb-rox-tdTomato* system was an efficient new tool to label proliferating cells.

### The Proliferating Ventricular Cardiomyocytes Were Efficiently Labeled in *Tnnt2-Dre* × *Aurkb-rox-tdTomato* Neonates

**FIGURE 2:**
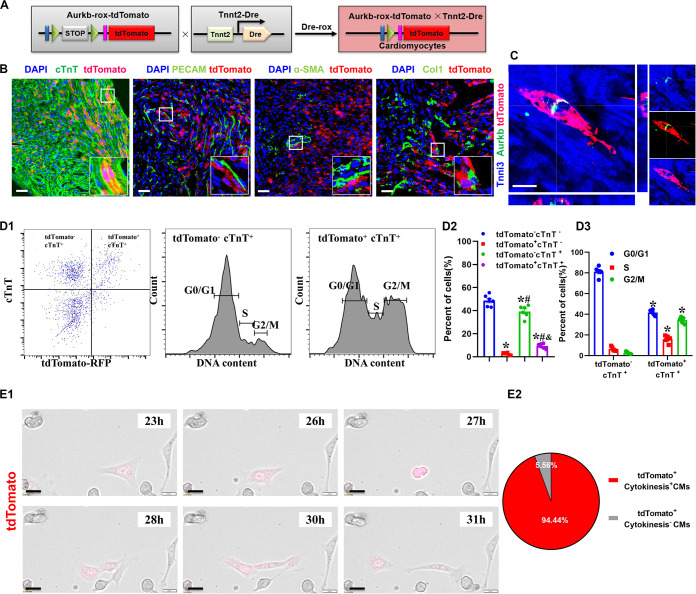
The proliferating ventricular cardiomyocytes were efficiently labeled in *Tnnt2-Dre* × *Aurkb-rox-tdTomato* neonates. **(A)** *Tnnt2-Dre* mice were used to cross with *Aurkb-rox-tdTomato* mice to label proliferating cardiomyocytes. **(B)** Immunostaining showed the coexpression of tdTomato with cTnT, PECAM, à-SMA, or Col1 on P1 *Tnnt2-Dre* × *Aurkb-rox-tdTomato* heart sections. Scale bar = 40 μm. **(C)** Immunostaining for tdTomato with Aurkb on P56 *Tnnt2-Dre* × *Aurkb-rox-tdTomato* heart sections. Scale bar = 20 μm. **(D)** Representative images of flow cytometry analysis **(D1)** and quantification of tdTomato^±^ cells **(D2)** and numbers in G1, S, G2/M phases **(D3)** from ventricles of P1 *Tnnt2-Dre* × *Aurkb-rox-tdTomato* mice. **(D2)** *n* = 6, *p < 0.05 vs. tdTomato^–^cTnT^–^; ^#^*p* < 0.05 vs. tdTomato^+^cTnT^–^; ^&^*p* < 0.05 vs. tdTomato^–^cTnT^+^. **(D3)** *n* = 5, *p < 0.05 vs. tdTomato^–^cTnT^+^. **(E)** Representative images **(E1)** and quantification **(E2)** of time-lapse imaging analysis showed cell proliferation *in vitro* of tdTomato^+^ cardiomyocytes from the ventricles of P1 *Tnnt2-Dre* × *Aurkb-rox-tdTomato* mice. Scale bar = 20 μm.

The accurate definition of cardiomyocyte renewal is the ability to replace lost cardiomyocytes by newly generated ones (Eschenhagen et al., 2017). This fact evokes the reassessment of complete proliferation potential in cardiomyocytes rather than only nuclear division. Therefore, we crossed *Tnnt2-Dre* mice with *Aurkb-rox-tdTomato* mice to label cardiomyocytes with complete proliferation potential (Figure 2A). A whole-cytoplasmic and specific expression of tdTomato was also confirmed in P56 *Tnnt2-Dre* × *Aurkb-rox-tdTomato* mice when marking cellular outline using WGA staining (Supplementary Figure 1B). In *Tnnt2-Dre* × *Aurkb-rox-tdTomato* mice (P1), immunostaining analysis showed that tdTomato was specifically expressed in cardiomyocytes, but not in non-cardiomyocytes, including endothelial cells, smooth muscle cells, and fibroblasts (Figure 2B). Aurkb protein expression was confirmed in adult tdTomato^+^ cardiomyocytes (P56, Figure 2C). Furthermore, cardiomyocytes were isolated from the ventricles of *Tnnt2-Dre* × *Aurkb-rox-tdTomato* mice (P1); cTnT labeling was carried out to exclude the impact of non-cardiomyocytes. Flow cytometry showed tdTomato was almost entirely expressed in cTnT^+^ cardiomyocytes; the percent of tdTomato^+^ cTnT^+^ cardiomyocytes (9.38% ± 0.90%) was significantly lower than tdTomato^–^ cTnT^+^ ones (39.27% ± 2.13%), and tdTomato^+^ cTnT^+^ cardiomyocytes exerted decreased cell numbers in G1 phase and increased cell numbers in the S and G2/M phases (Figure 2D). The live time-lapse imaging observation showed that 94.44% of the tdTomato^+^ cardiomyocytes underwent a complete proliferation process (Figure 2E and Supplementary Video 2). These results suggested that the *Tnnt2-Dre* × *Aurkb-rox-tdTomato* system was an efficient tool for identifying and analyzing the live proliferating ventricular cardiomyocytes, at least in neonates.

### The Variant Proliferation Potential During Early Postnatal Development

**FIGURE 3:**
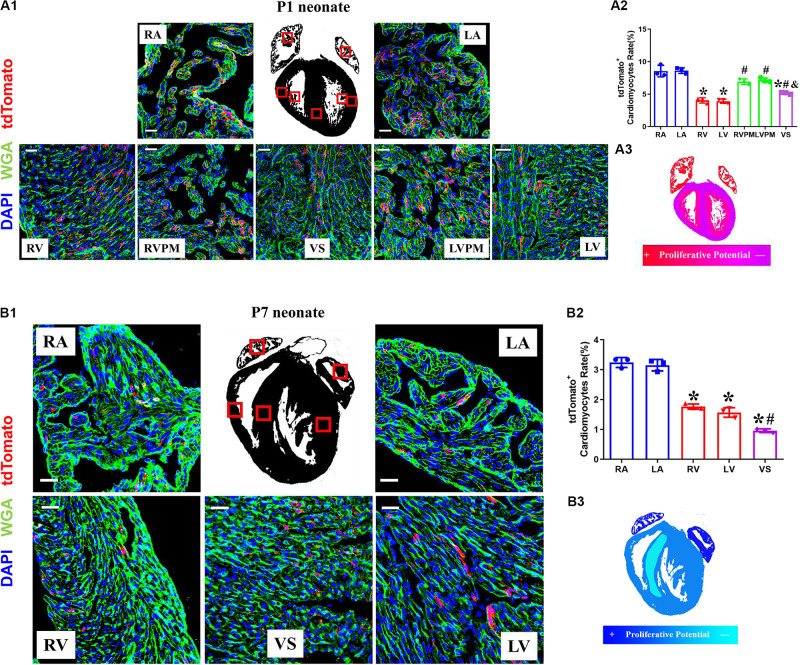
The variant proliferation potential during early postnatal development. **(A)** Representative images **(A1)** and quantification **(A2)** of immunostaining for tdTomato with wheat germ agglutinin (WGA) staining on P1 *Tnnt2-Dre* × *Aurkb-rox-tdTomato* heart sections in different regions, RA, right atrium; LA, left atrium; RV, right ventricle; LV, left ventricle; RVPM, right ventricular papillary muscle; LVPM, left ventricular papillary muscle; VS, ventricular septum. Scale bar = 40 μm, *n* = 3, *p < 0.05 vs. atrium; ^#^*p* < 0.05 vs. ventricular; ^&^*p* < 0.05 vs. ventricular papillary muscle. **(A3)** Schematic figure showing variant cytokinesis capacity on P1 *Tnnt2-Dre* × *Aurkb-rox-tdTomato* heart sections in different regions. **(B)** Representative images **(B1)** and quantification **(B2)** of immunostaining for tdTomato with WGA staining on P7 *Tnnt2-Dre* × *Aurkb-rox-tdTomato* heart sections in different regions, RA, right atrium; LA, left atrium; RV, right ventricle; LV, left ventricle; VS, ventricular septum. Scale bar = 40 μm, *n* = 3, *p < 0.05 vs. atrium; ^#^*p* < 0.05 vs. ventricular. **(B3)** Schematic figure showing variant cytokinesis capacity on P7 *Tnnt2-Dre* × *Aurkb-rox-tdTomato* heart sections in different regions.

The neonatal mammalian heart is capable of regeneration, and this regenerative capacity is lost within the first week of life (Porrello et al., 2011). We detected the quantification and distribution of cardiomyocytes with complete proliferation potential during early postnatal development in *Tnnt2-Dre* × *Aurkb-rox-tdTomato* mice. Immunostaining was performed on P1 and P7 heart sections. In P1 hearts, atrial myocardium showed higher tdTomato^+^ rates than ventricle, but no significant difference was observed between the left and right atrium or ventricle (P1: RA: 8.56% ± 0.51%; LA: 8.62% ± 0.25%; RV: 4.05% ± 0.24%; LV: 3.92% ± 0.18%; RVPM: 6.93% ± 0.25%; LVPM: 7.17% ± 0.20%; VS:5.16% ± 0.08%). Distinct papillary muscle can be seen in P1 ventricles where more tdTomato^+^ cardiomyocytes were detected than compacted ventricles, and tdTomato^+^ rate in ventricular septum was higher than compacted ventricle (Figure 3A). In addition, fewer tdTomato^+^ cardiomyocytes were found in P7 hearts than P1 ones (P7: RA: 3.24% ± 0.10%; LA: 3.15% ± 0.11%; RV: 1.77% ± 0.05%; LV: 1.57% ± 0.09%; VS: 0.96% ± 0.04%), suggesting stronger proliferative capacity in P1 cardiomyocytes (two to three 3 times over P7 in ventricles). P7 hearts showed the same distributional characteristics of tdTomato^+^ cardiomyocytes except that the tdTomato^+^ rate in ventricular septum was lower than the compacted ventricle (Figure 3B).

EdU pulse was performed at P3 and P5 and then detected at P7 in *Tnnt2-Dre* × *Aurkb-rox-tdTomato* mice (Supplementary Figure 3A). There were significantly increased EdU^+^tdTomato^+^ cardiomyocytes compared with EdU^–^tdTomato^+^ ones, suggesting most proliferating cardiomyocytes were monitored by tdTomato labeling (Supplementary Figure 3B). The tdTomato^+^ rates of non-cardiomyocytes were also identified in *CAG-Dre* × *Aurkb-rox-tdTomato* mice; there were decreased tdTomato^+^ endothelial and smooth muscle cells in P7 hearts. In contrast, no significant tdTomato^+^ rates of fibroblasts were changed between P1 and P7 hearts (Supplementary Figure 4). The time- and region-dependent variant proliferation potential might be associated with different cavity pressure or tightness of cell arrangement.

### The Analysis of tdTomato^+^ Cardiomyocytes With Different Ploidy

**FIGURE 4:**
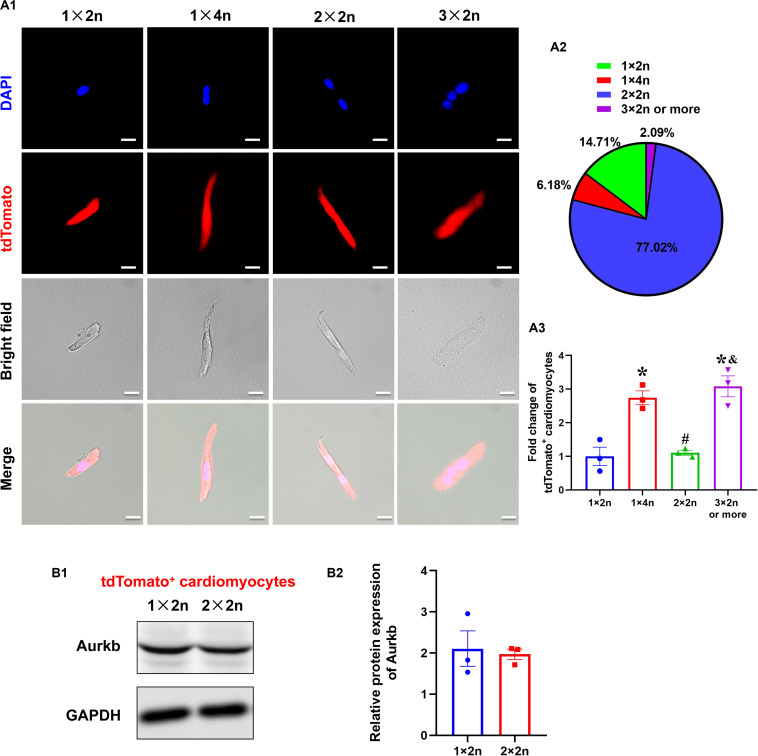
The analysis of tdTomato^+^ cardiomyocytes with different ploidy. **(A,A1)** Immunostaining for tdTomato with DAPI on P7 isolated rod-shaped cells of *Tnnt2-Dre* × *Aurkb-rox-tdTomato* ventricles. Scale bar = 20 μm. **(A2)** Quantification of nucleation and ploidy proportion on P7 isolated rod-shaped cells of *Tnnt2-Dre* × *Aurkb-rox-tdTomato* ventricles. **(A3)** Relative fold change of tdTomato^+^ proportion on P7 isolated rod-shaped cells of *Tnnt2-Dre* × *Aurkb-rox-tdTomato* hearts in each nucleation/ploidy proportion. *n* = 3, *p < 0.05 vs. 1 × 2n; ^#^*p* < 0.05 vs. 1 × 4n; ^&^*p* < 0.05 vs. 2 × 2n. **(B)** Representative images **(B1)** and quantification **(B2)** of Aurkb protein expression in sorting diploid (1 × 2n) and tetraploid (2 × 2n) tdTomato^+^ cardiomyocytes from P7 *Tnnt2-Dre* × *Aurkb-rox-tdTomato* ventricles, analyzed by Western blotting. GAPDH was used as control. *n* = 3.

Previous studies suggested that proliferating cardiomyocytes were predominantly mononucleated or diploid (Hirose et al., 2019). The mouse heart is capable of regeneration for the first postnatal week when multinucleation or polyploidization occurs in cardiomyocytes. There are mainly mononuclear cardiomyocytes that are the source of cardiac regeneration in P1 heart; however, accumulating evidence has indicated that mononucleated and multinucleated adult cardiomyocytes constitute a transcriptionally homogenous cell population and cytokinesis rates through single-cell analysis (Yekelchyk et al., 2019) and live-cell imaging (Wang et al., 2017). To explore if there is reserved proliferative capacity of mononucleated cardiomyocytes when most cardiomyocytes have become binucleated/multinucleated, an analysis of isolated cardiomyocytes from P7 neonatal ventricles identified the proportion of tdTomato^+^ cardiomyocytes with different ploidy (diploid: 1 × 2n, 14.71%; tetraploid: 1 × 4n, 6.18% and 2 × 2n, 77.02%; polyploid: 3 × 2n or more, 2.09%; Figures 4A1,A2). Interestingly, the normalized tdTomato^+^ rates were comparable in tetraploid (2 × 2n) and diploid cardiomyocytes, whereas the rates were higher in tetraploid (1 × 4n) and polyploid cardiomyocytes (Figure 4A3). Moreover, there was no significant difference between diploid (1 × 2n) and tetraploid (2 × 2n) tdTomato^+^ cardiomyocytes in the protein expression of Aurkb (Figure 4B). These results indicate that mononucleated cardiomyocytes might not possess significantly higher proliferating potential than other cardiomyocytes when most cardiomyocytes have become postmitotic.

### The Proliferation Potential of Cardiomyocytes After Apex Resection or Myocardial Infarction

**FIGURE 5:**
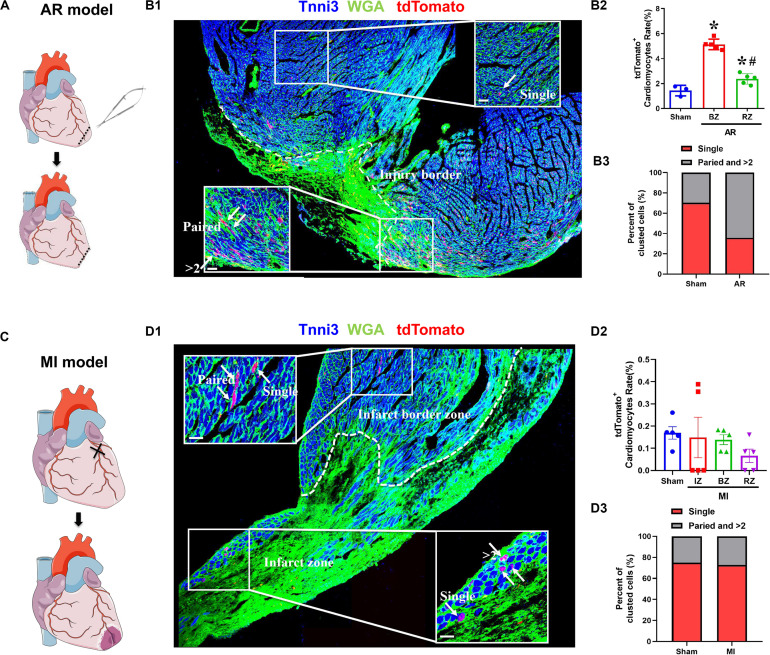
The proliferation potential of cardiomyocytes after apex resection or myocardial infarction. **(A)** Schematic figure showing the procedure of apex resection (AR) on neonatal hearts. **(B)** Representative image **(B1)** and quantification **(B2)** of tdTomato^+^ cardiomyocytes rate in different regions of apex resection or sham hearts; apex resection was conducted at P1, and hearts were harvested at P7, BZ, border zone; RZ, remote zone; AR, apex resection. Scale bar = 40 μm, *n* = 5, *p < 0.05 vs. sham; ^#^*p* < 0.05 vs. BZ. **(B3)** Single, paired, and >2 clustered tdTomato^+^ cardiomyocytes of post-AR hearts were analyzed. **(C)** Schematic figure showing the procedure of myocardial infarction (MI) on adult hearts. **(D)** Representative image **(D1)** and quantification **(D2)** of tdTomato^+^ cardiomyocytes rate in different regions of MI or sham hearts. MI was conducted at P56, and hearts were harvested at P70, IZ, infarct zone; BZ, border zone; RZ, remote zone. Scale bar = 40 μm, *n* = 5. **(D3)** Single, paired, and >2 clustered tdTomato^+^ cardiomyocytes of post-MI hearts were analyzed.

We determined the proliferation potential of cardiomyocytes in injured neonatal and adult *Tnnt2-Dre* × *Aurkb-rox-tdTomato* mice. In a neonatal mouse model of apex resection (Figure 5A), the tdTomato^+^ cardiomyocytes were significantly increased and enriched at injury border zone (Figure 5B), suggesting that proliferation was stimulated. It has been esteemed that MI could stimulate cardiomyocyte proliferation in adults, but it is complicated with multinucleation or polyploidization. A previous study using a reporter tool of chromosome segregation revealed that MI could not elevate cytokinesis rate (Ali et al., 2014). Consistent with previous results, there were no increased tdTomato^+^ cardiomyocytes after MI in adults using our system (Figures 5C,D). To exclude possible false-positive labeling of multinucleation/polyploidization, single, paired, and >2 tdTomato^+^ clustered cardiomyocytes were distinguished (the latter two forms are more likely to experience cell division). Compared with sham, there were significantly increased paired and >2 tdTomato^+^ clustered cardiomyocytes after apex resection in neonates, whereas no increased paired and >2 tdTomato^+^ clustered cardiomyocytes were observed after MI in adults (Figures 5B3,D3).

## Discussion

In the present study, we generated an Aurkb transcription–driven un-fusion red fluorescent protein (*Aurkb-rox-tdTomato*) under the control of endogenous Aurkb locus in mouse heart in a Dre-dependent manner, which represented a whole-cytoplasmic and distinguishing readout of complete proliferation potential in cardiomyocytes or other cell types of interest. Using cardiomyocyte-specific marker cardiac troponin T2 (Tnnt2)-Dre mice, we described the time- and region-dependent variant proliferation potential during the heart’s early postnatal development. Additionally, the analysis of tdTomato^+^ cardiomyocytes at postnatal 7 days with different ploidy indicated that mononucleated cardiomyocytes might not possess significantly higher proliferating potential than other cardiomyocytes. After apex resection in the neonatal mammalian heart, which is capable of regeneration, the tdTomato^+^ cardiomyocytes were stimulated and enriched at injury border zone in neonates, while there were no increased tdTomato^+^ cardiomyocytes after MI in adult hearts.

The accurate definition of cardiomyocyte renewal is the ability to replace lost cardiomyocytes by new ones, which is distinct from nuclear division, giving rise to multinucleation or DNA duplication without nuclear division resulting in polyploid nuclei (Eschenhagen et al., 2017). Traditional staining of Ki67, BrdU/EdU, and PHH3 poorly differentiates authentic cell division from endoreduplication, cytokinetic mitosis, or DNA repair. Owing to tissue complexity with a variety of non-cardiomyocytes, traditional cardiomyocyte-specific staining of the above markers is exceptionally challenging, and BrdU/EdU can also label cells that have exited cell cycle (Hesse et al., 2012).

Accordingly, recent efforts have achieved significant progress in discerning cardiomyocyte renewal. Based on tissue-specific FUCCI (fluorescent ubiquitination-based cell cycle indicator) indicators, the dual-color visualization provides a more refined approach to label the cardiomyocytes in G1 or S/G2/M phases (Abe et al., 2013; Goto et al., 2015; Alvarez et al., 2019). One advantage of our Aurkb reporter–based mouse system is relying on a single fluorescence marker and with better specificity in cells with complete proliferation potential, leaving other fluorescence channels available for additional markers. Recently described mosaic analysis with double markers (MADM) system can indelibly and uniquely label two daughter cells of a dividing cell as GFP^+^ or RFP^+^ because of restructuring of homologous chromosomes sister chromatids (Ali et al., 2014). However, there are two kinds of separation of homologous recombination; the MADM system underestimates at least 50% of cytokinesis cells, and YFP^+^ or unlabeled cells can also possibly undergo authentic cell division. In addition, the MADM system is designed to label post-cytokinesis, which is not conducive to the sorting and comparison of proliferating and non-proliferating cardiomyocytes. Another analogous proliferation-tracing system is based on fusing enhanced green fluorescent protein (eGFP) to the scaffolding protein anillin, which is a component of the contractile ring precisely localizing to the cleavage furrow during M-phase (Hesse et al., 2012, 2018). The limitation of an anillin-eGFP system is that the fluorescent signal is concentrated as small dots between cells, which is challenging to sort isolated eGFP^+^ cardiomyocytes and distinguish from non-specific staining. Admittedly, our Aurkb reporter–based mouse system overestimated the percentage of cell proliferation because of Aurkb expression between some dividing nuclei.

Using the Aurkb-tdTomato system, we discovered that atrium cardiomyocytes showed more energetic proliferative potential than those of ventricle. Recent studies have demonstrated that cardiomyocyte proliferation can be locally stimulated by an acute increase or decrease of ventricular pressure (Unno et al., 2019). Furthermore, mature cardiomyocytes reenter the cell cycle and generate new cardiomyocytes through a three-step process as dedifferentiation, proliferation, and redifferentiation, and the main manifestations of dedifferentiation are sarcomere depolymerization and decreased contractility (Wang et al., 2017). Therefore, we assumed that the atrium’s stronger proliferative potential was explained by low pressure and weak contractility. Intriguingly, in our study, cardiomyocytes in different regions of the same ventricle still showed different proliferative potentials; distinct papillary muscle revealed higher tdTomato^+^ cardiomyocytes than compacted ventricle, which might be relative to the stiffness of cardiomyocytes arrangement.

Based on the new definition of cardiomyocyte renewal, we have focused on the controversial issue of whether cardiomyocyte renewal rates were higher after injury than under normal conditions. Consistent with other reported proliferation-tracing systems (Hesse et al., 2012; Ali et al., 2014), we provided further evidence that cardiomyocytes’ proliferation potential was stimulated after apex resection neonates, while there were no increased tdTomato^+^ cardiomyocytes after MI in adults. Owing to the overestimated limitation of the Aurkb-tdTomato system, we distinguished tdTomato^+^ cardiomyocytes after MI as single, paired, and >2 tdTomato^+^ clusters, and no increased paired and >2 tdTomato^+^ clustered cardiomyocytes were observed after MI.

This study highlights the advantages of an Aurkb–specific proliferation reporter to study cardiomyocyte renewal regulation and general utility in other cell types. Unlike previous reporter tools, we constructed a new balance between sensitivity and specificity in cell proliferation assessment. This system provided a new perspective to address cardiomyocyte renewal in different heart regions of the early postnatal period and a potential tool to investigate cardiac regeneration in the adult heart. Additionally, it should also be noted that there have been utilized potential of the Aurkb-tdTomato system in the development and regeneration of other organs and in the context of oncotherapy.

## Data Availability Statement

The data that support the findings of this study and study materials, as well as experimental procedures and protocols, are available from the corresponding authors on reasonable request.

## Ethics Statement

The animal study was reviewed and approved by Institutional Animal Care and Use Committee at Third Military Medical University.

## Author Contributions

WF and WW designed the study, performed experiments, and analyzed the data. WF, QL, LL, YS, and AZ bred the mice and performed experiments. CZ and WW conceived and supervised the study, analyzed the data, and wrote the manuscript. All authors contributed to the article and approved the submitted version.

## Conflict of Interest

The authors declare that the research was conducted in the absence of any commercial or financial relationships that could be construed as a potential conflict of interest.

## Supplementary Material

Supplementary Figure 1 **(A)** Immunostaining for tdTomato with cTnT staining on P4 *Aurkb-rox-tdTomato* heart sections with and without crossing by *CAG-Dre*. Scale bars = 400 μm. **(B)** Immunostaining for tdTomato with WAG staining on P56 *Aurkb-rox-tdTomato* heart sections with and without crossing by *CAG-Dre or Tnnt2-Dre*. Scale bars = 40 μm.

Supplementary Figure 2 **(A)** Quantification of Aurkb mRNA expression in P1 ventricles from wild type, *CAG-Dre*, *Tnnt2-Dre*, *Aurkb-rox-tdTomato*, *CAG-Dre* × *Aurkb-rox-tdTomato*, and *Tnnt2-Dre* × *Aurkb-rox-tdTomato* mice, analyzed by qPCR. GAPDH was used as control. *N* = 3. **(B)** Representative images **(B1)** and quantification **(B2)** of Aurkb protein expression in sorting tdTomato^+^ and tdTomato^–^ cells from P1 *CAG-Dre* × *Aurkb-rox-tdTomato* ventricles, analyzed by western blotting. GAPDH was used as control. *N* = 6, ^∗^*p* < 0.05 vs. tdTomato^+^ cells.

Supplementary Figure 3 **(A)** *Tnnt2-Dre* × *Aurkb-rox-tdTomato* mice were injected intraperitoneally with EdU at 100 μg per animal in P3 and P5. The hearts were harvested at P7. **(B)** Representative images **(B1)** and quantification **(B2)** of immunostaining for tdTomato with wheat germ agglutinin (WGA), EdU, and Tnni3 on P7 *Tnnt2-Dre* × *Aurkb-rox-tdTomato* heart sections after injecting intraperitoneally with EdU at P3 and P5. Scale bars = 40 μm, *N* = 5, ^∗^*p* < 0.05 vs. EdU^+^tdTomato^–^ cardiomyocytes, ^#^*p* < 0.05 vs. EdU^–^tdTomato^+^ cardiomyocytes. **(B3)** Schematic figure showing the overlap between labeled cardiomyocytes with EdU and tdTomato.

Supplementary Figure 4 Representative images **(A1,B1,C1)** and quantification **(A2,B2,C2)** of the co-expression of tdTomato with Col1 **(A)**, PECAM **(B)** and a-SMA **(C)** on P1 and P7 *CAG-Dre* × *Aurkb-rox-tdTomato* heart sections. Scale bars = 40 μm, *N* = 5, ^∗^*p* < 0.05 vs. P1.
