# Supplementary material for: An Aurora Kinase B–Based Mouse System to Efficiently Identify and Analyze Proliferating Cardiomyocytes
Source: Front Cell Dev Biol. 2020 Oct 7;8:570252. doi: 10.3389/fcell.2020.570252 (PMC7575716; doi:10.3389/fcell.2020.570252)
Supplement: Supplementary file 1 [file Table_1.DOCX]

| **Supplemental Table 1** | |
| --- | --- |
| Aurkb-rox-tdTomato | ATGTTTCAGGTTCAGGGGGAGGTG(5'-3') |
|  | GCATCTCTTGCCTTCGCTGTTG(5'-3') |
|  | TGGATTCTTACTTCCCACCGC(5'-3') |
|  | TTGGTCACCTTCAGCTTGGC(5'-3') |
| CAG-Dre | ACTCCTTGCCGATGTTCCTCAG(5'-3') |
|  | TTGTCCCAAATCTGGCGGAG(5'-3') |
| Tnnt2-Dre | GCTGCCTTGCTGTGTTGTTCAG(5'-3') |
|  | ACTCCTTGCCGATGTTCCTCAG(5'-3') |
|  | TGTGTATTCCCAAAGTCCCCAG(5'-3') |
| Aurkb | CAGAAGGAGAACGCCTACCC(5'-3') |
|  | GAGAGCAAGCGCAGATGTC(5'-3') |
| GAPDH | AGGTCGGTGTGAACGGATTTG(5'-3') |
|  | TGTAGACCATGTAGTTGAGGTCA(5'-3') |
