# Supplementary figures and images for: An Aurora Kinase B–Based Mouse System to Efficiently Identify and Analyze Proliferating Cardiomyocytes
Source: Front Cell Dev Biol. 2020 Oct 7;8:570252. doi: 10.3389/fcell.2020.570252 (PMC7575716; doi:10.3389/fcell.2020.570252)

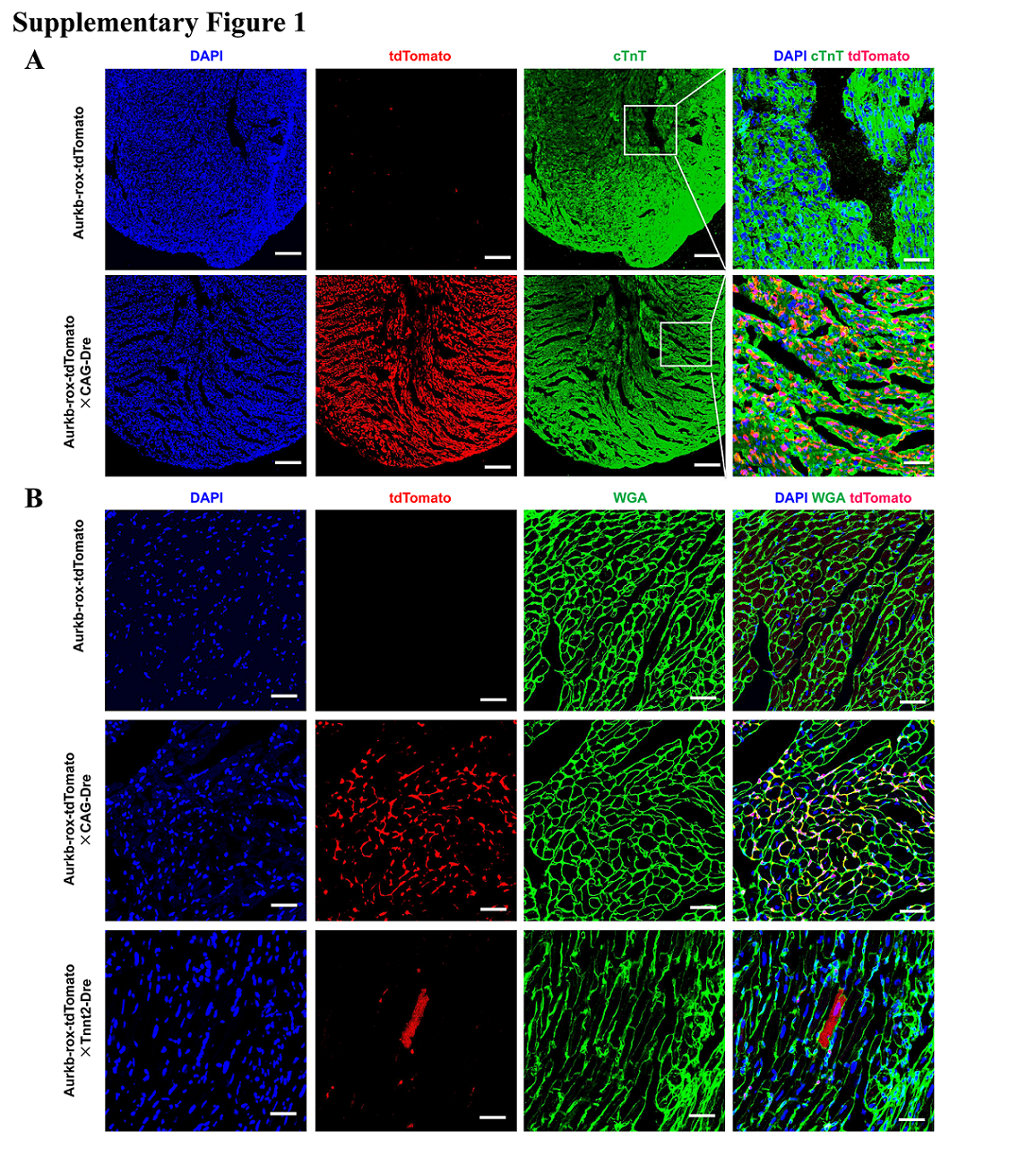

Supplement: Supplementary Figure 1 — (A) Immunostaining for tdTomato with cTnT staining on P4 Aurkb-rox-tdTomato heart sections with and without crossing by CAG-Dre. Scale bars = 400 μm. (B) Immunostaining for tdTomato with WAG staining on P56 Aurkb-rox-tdTomato heart sections with and without crossing by CAG-Dre or Tnnt2-Dre. Scale bars = 40 μm. [file Image_1.tif]

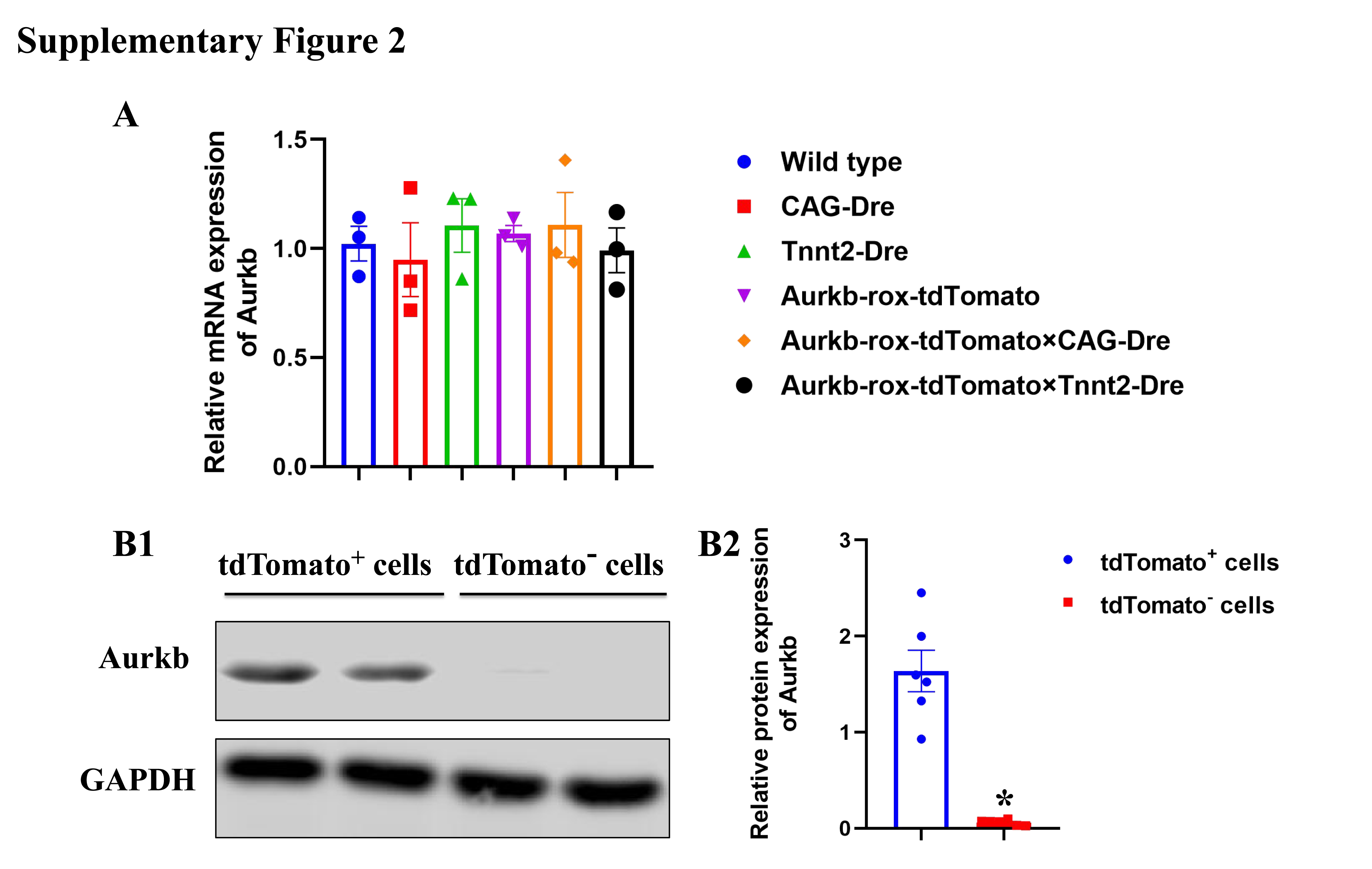

Supplement: Supplementary Figure 2 — (A) Quantification of Aurkb mRNA expression in P1 ventricles from wild type, CAG-Dre, Tnnt2-Dre, Aurkb-rox-tdTomato, CAG-Dre × Aurkb-rox-tdTomato, and Tnnt2-Dre × Aurkb-rox-tdTomato mice, analyzed by qPCR. GAPDH was used as control. N = 3. (B) Representative images (B1) and quantification (B2) of Aurkb protein expression in sorting tdTomato+ and tdTomato– cells from P1 CAG-Dre × Aurkb-rox-tdTomato ventricles, analyzed by western blotting. GAPDH was used as control. N = 6, ∗p < 0.05 vs. tdTomato+ cells. [file Image_2.tif]

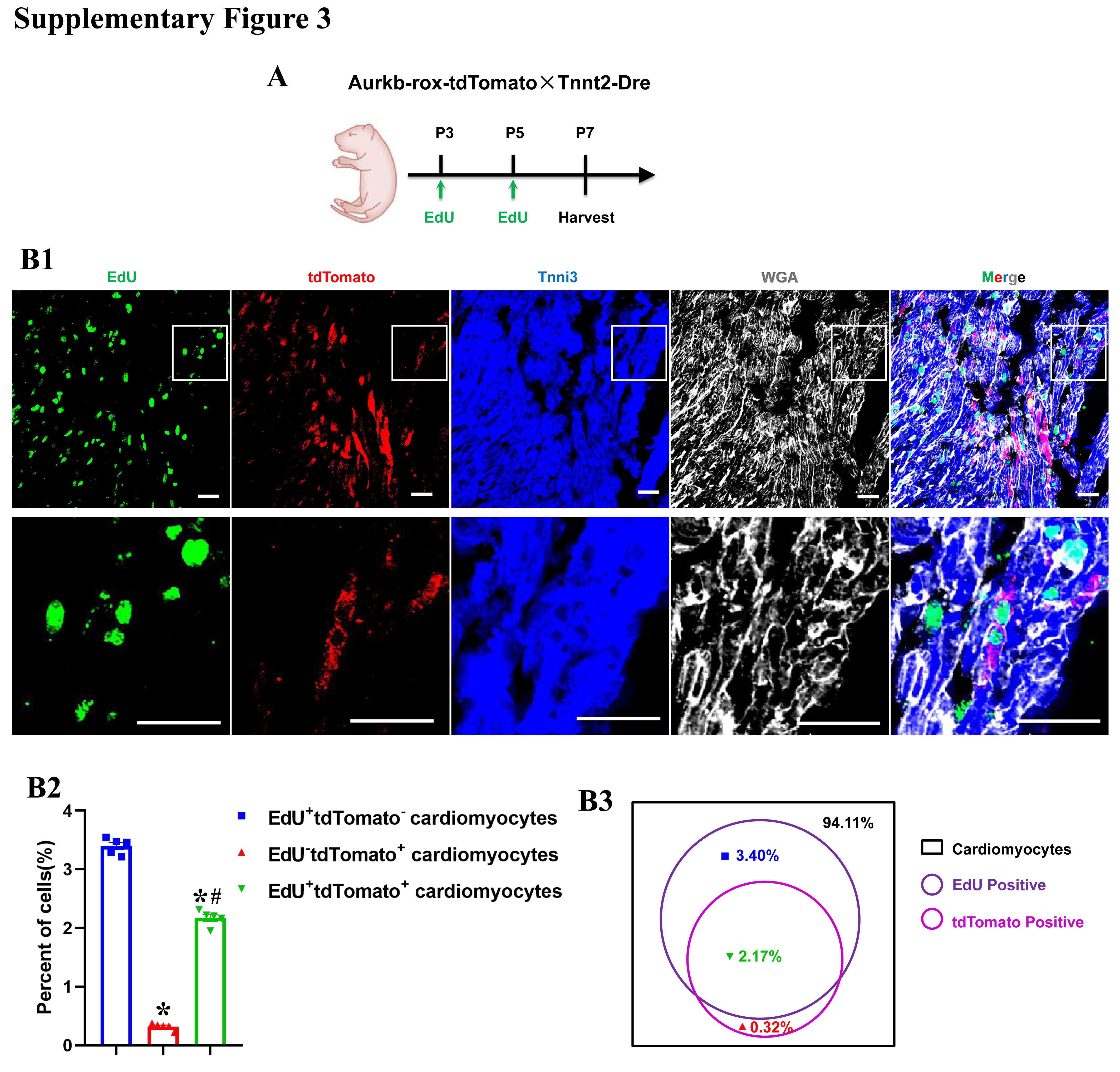

Supplement: Supplementary Figure 3 — (A) Tnnt2-Dre × Aurkb-rox-tdTomato mice were injected intraperitoneally with EdU at 100 μg per animal in P3 and P5. The hearts were harvested at P7. (B) Representative images (B1) and quantification (B2) of immunostaining for tdTomato with wheat germ agglutinin (WGA), EdU, and Tnni3 on P7 Tnnt2-Dre × Aurkb-rox-tdTomato heart sections after injecting intraperitoneally with EdU at P3 and P5. Scale bars = 40 μm, N = 5, ∗p < 0.05 vs. EdU+tdTomato– cardiomyocytes, #p < 0.05 vs. EdU–tdTomato+ cardiomyocytes. (B3) Schematic figure showing the overlap between labeled cardiomyocytes with EdU and tdTomato. [file Image_3.jpg]

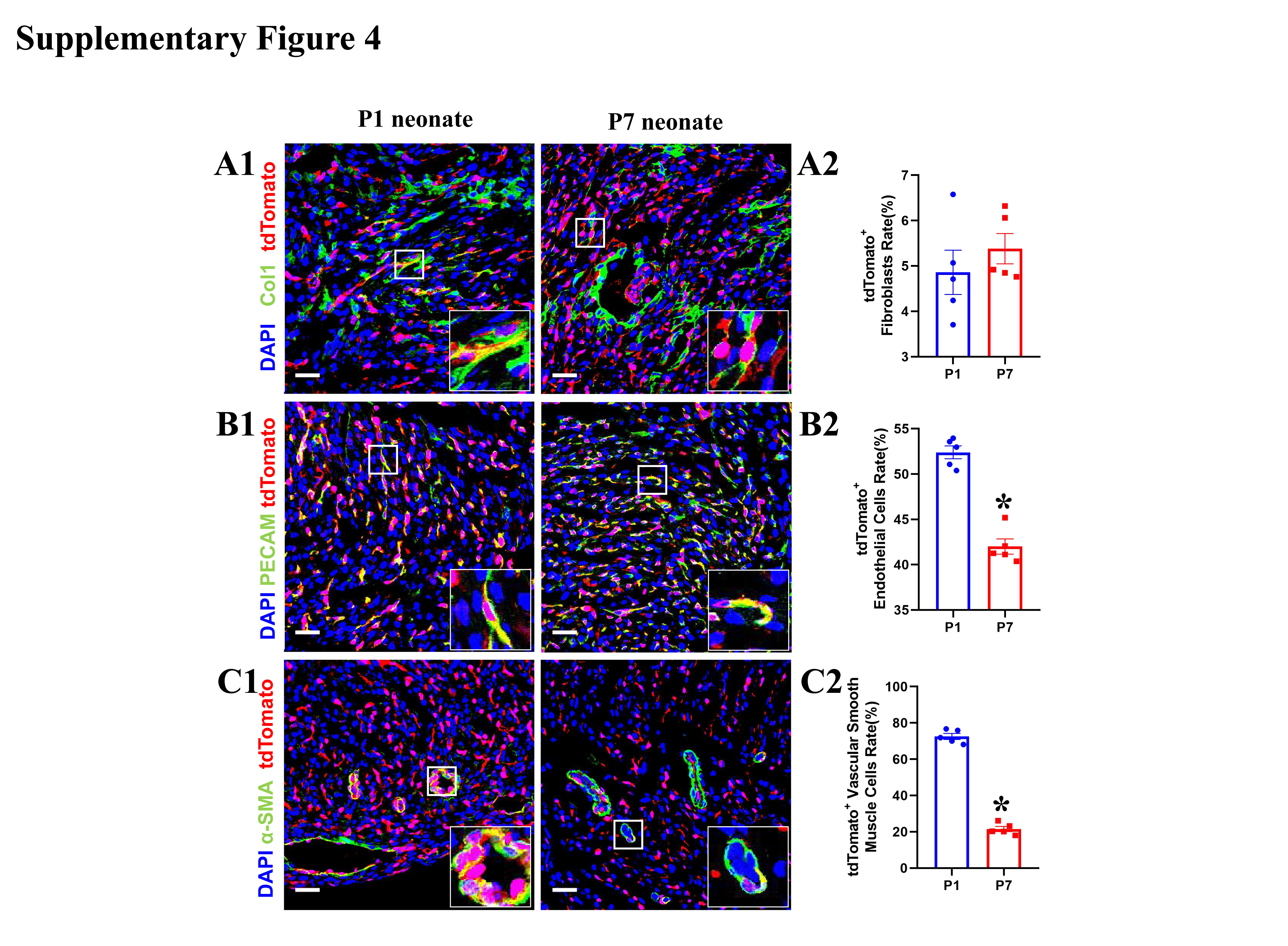

Supplement: Supplementary Figure 4 — Representative images (A1,B1,C1) and quantification (A2,B2,C2) of the co-expression of tdTomato with Col1 (A), PECAM (B) and a-SMA (C) on P1 and P7 CAG-Dre × Aurkb-rox-tdTomato heart sections. Scale bars = 40 μm, N = 5, ∗p < 0.05 vs. P1. [file Image_4.tif]
